# Liposome Deformation Induced by Membrane-Binding Peptides

**DOI:** 10.3390/mi14020373

**Published:** 2023-02-02

**Authors:** Kayano Izumi, Chihiro Saito, Ryuji Kawano

**Affiliations:** Department of Biotechnology and Life Science, Tokyo University of Agriculture and Technology, Tokyo 184-8588, Japan

**Keywords:** liposome deformation, peptide, membrane capacitance

## Abstract

This paper presents an investigation of liposome deformation and shape distortion using four membrane-binding peptides: TAT and C105Y as cell-penetrating peptides (CPPs), and melittin and ovispirin as antimicrobial peptides (AMPs). Liposome deformation was monitored utilizing fluorescent microscopy, while the binding of peptides to the DOPC membrane was estimated through capacitance measurements. The degree of liposome deformation and shape distortion was found to be higher for the CPPs compared to the AMPs. Additionally, it was observed that C105Y did not induce liposome rupture, unlike the other three peptides. We propose that these variations in liposome distortion may be attributed to differences in secondary structure, specifically the presence of an α-helix or random coil. Our studies offer insight into the use of peptides to elicit control of liposome architecture and may offer a promising approach for regulating the bodies of liposomal molecular robots.

## 1. Introduction

Cell-sized liposomes are frequently employed in the study of model cells or cell membranes. They are also utilized in the field of synthetic biology for encapsulating materials, particularly in the realm of “Build-a-Cell” research [1,2]. For instance, various reaction networks utilizing enzymatic and chemical reactions, as well as protein expression, have been implemented within liposomes [3]. Building upon the technologies of synthetic biology, the field of molecular robotics has emerged as a subdiscipline, which shares similarities with “Build-a-Cell” research but addresses challenges from an engineering perspective [4,5,6]. A molecular “robot” can be defined as a micro-robot consisting of molecules and comprising three essential components: a sensor for perception, a calculator for intelligence, and actuators for motor function. These functionalities are integrated within a micron-sized body surrounded by a lipid membrane. In 2017, Sato et al. reported the prototype of a sophisticated molecular robot, in which they developed an amoeba-type robot with light-induced DNA clutches for sensors and kinesin-microtubule proteins as actuators, all integrated within a cell-sized liposome. Light irradiation serves as a trigger for the release of signaling molecules and engagement of the DNA clutches, leading to a change in the shape of the liposome [7].

The control of liposome shape has been a topic of investigation in molecular robotics, as deformability is a necessary characteristic for movement within micro and crowded environments, such as within living organisms or soil. For example, deformable liposomes containing surfactants have been shown to permeate human skin, enabling the transdermal transport of drugs [8,9]. Several approaches have been proposed to deform liposomes, including the use of surfactants [10,11], proteins [3,12,13], and peptides [14,15,16,17,18,19,20]. The use of fatty acid surfactants, such as oleate, has been shown to induce the growth, division, and budding of phospholipid or oleate liposomes [10]. Additionally, surfactants have been demonstrated to induce shape deformation in fatty acid vesicles, with deformation dependent on the type of surfactant; for example, the size of vesicles rapidly increases with the addition of Triton X-100, and sodium dodecyl sulfate induces changes in membrane curvature [11]. Proteins have also been shown to induce deformation in liposomes through encapsulated polymerization of cytoskeletal components [12]. For example, Furusato et al. expressed several proteins involved in the formation of the Z-ring in bacterial cytokinesis and induced liposome deformation through their presence [3]. Remarkable deformation from spherical to other structures was observed under certain conditions, such as in the presence of FtsZ and ZipA. In the case of using peptides, liposome budding was induced by a partial sequence derived from matrix-2 (M2), a component of the influenza virus [20]. Additionally, various researchers have reported the use of a venom toxin, melittin, to induce liposome deformation. As methodologies for peptide design and synthesis have been significantly advanced in recent decades, peptides have emerged as a potential material for the transformation of molecular robots [21].

We here investigated 1,2-dioleoyl-*sn*-glycero-3-phosphocholine (DOPC) liposome deformation induced by the following four peptides which are known as antimicrobial peptides (AMPs) and cell-penetrating peptides (CPPs) which can spontaneously bind to bilayer lipid membranes:

(1) Human immunodeficiency virus (HIV)-1 tat (TAT, YGRKKRRQRRR, 11 amino acids) is derived from 47–57 amino acids of the HIV-1 transcriptional activator [22,23,24,25].

(2) C105Y (CSIPPEVKFNKPFVYLI, 17 amino acids) is a synthetic peptide based on 359–374 residues of α1-antitrypsin [24,26].

(3) Melittin (GIGAVLKVLTTGLPALISWIKRKRQQ, 26 amino acids) is derived from Apis mellifera (a honey bee), and some studies have reported liposome deformation using this peptide [19,27,28,29,30,31,32].

(4) Ovispirin (KNLRRIIRKIIHIIKKYG, 18 amino acids) is derived from the N-terminal 18 amino acids of SMAP-29 [33,34].

There have been numerous reports on the secondary structures of the peptides at the PC membrane including DOPC. The CPPs, such as TAT and C105Y, have been proposed to exhibit a tendency towards random coil structures [24,25], whereas the AMPs (melittin and ovispirin) have been suggested to primarily adopt an α-helical structure [28,29,33,34]. In this study, we hypothesized that they may also exhibit random coil and amphipathic α-helix structures.

To investigate liposome deformation induced by these peptides, we employed fluorescent microscopy and measured membrane capacitance. The latter approach allows for the determination of changes in membrane properties, such as surface area, permittivity, and thickness, resulting from peptide binding. Based on our results, we evaluated the effectiveness of the peptides in inducing liposome deformation.

## 2. Materials and Methods

### 2.1. Experimental Materials

In this study, the following chemicals were used: 1,2-dioleoyl-*sn*-glycero-3-phosphocholine (DOPC; Avanti Polar Lipids, Birmingham, AL, USA), 1,2-dioleoyl-*sn*-glycero-3-phosphoethanolamine-N-(lissamine rhodamine B sulfonyl) (ammonium salt) (Rhodamine PE; Avanti Polar Lipids, Birmingham, AL, USA), liquid paraffin (FUJIFILM Wako Pure Chemical Corporation, Osaka, Japan), calcein (Sigma-Aldrich Co, LCC., St. Louis, MO, USA), glucose (FUJIFILM Wako Pure Chemical Corporation, Osaka, Japan), sucrose (FUJIFILM Wako Pure Chemical Corporation, Osaka, Japan), *n*-decane (FUJIFILM Wako Pure Chemical Corporation, Osaka, Japan), 3-morpholinopropane-1-sulfonic acid (MOPS, Nacalai Tesque, Kyoto, Japan), potassium chloride (KCl, Nacalai Tesque, Kyoto, Japan). As peptides, we used C105Y (GenScuript, Piscataway, NJ, USA), melittin (synthesized and purified as powder), ovispirin (KareBay Biochem, Inc., Monmouth Junction, NJ, USA), and TAT (BACHEM, Bubendorf, Switzerland). Peptides were stored at −20 °C. For use, samples were diluted to their designated concentration using a buffered electrolyte solution and stored at 4 °C.

### 2.2. Preparation of Giant Liposomes Using the Droplet Transfer Method

Liposomes of 98:2 DOPC/Rhodamine PE (mol %) were prepared using droplet transfer. [35] First, 12.1 µL of 32.6 mM lipid mixture dissolved in chloroform was poured into a glass vial and evaporated under a flow of nitrogen until a lipid film formed at the bottom of the vial. The vial was then put in a desiccator (AZ ONE Corporation, Osaka, Japan) for more than three hours to completely remove the remaining chloroform. Next, 300 µL of liquid paraffin was added to the vial and stirred with the lipids using a 40 kHz ultrasonic cleaner MSC-2 (AZ ONE Corporation, Osaka, Japan) at 50 °C for an hour. Then, a 20 µL liquid mixture of 0.5 M sucrose and 0.5 mM calcein dissolved in MilliQ ultra-pure water (MQ) was added to the vial, and the contents were mixed by tapping 40 times, to form water-oil (W/O) emulsions. 150 µL of the content was slowly added to 150 µL of 0.5 M glucose dissolved in MQ in a 500 µL polycarbonate centrifuge tube and centrifuged at 8000× *g* for 5 min with centrifuge CT15E (Hitachi Koki Co. Ltd., Tokyo, Japan). Giant liposomes were precipitated at the bottom of the tube by the oil-to-water phase transfer of the W/O emulsions. The pellet of the liposomes was extracted, resuspended into a new centrifuge tube with 300 μL of 0.5 M glucose dissolved in MQ, and centrifuged again at 6000× *g* for 10 min to remove small lipid aggregates. After centrifuging twice, the pellets of liposomes were loaded into a new centrifuge tube with 100 μL of 0.5 M glucose dissolved in MQ. All preparations were conducted at room temperature (RT) unless otherwise specified. Liposomes were observed using an IX71 fluorescence microscope (OLYMPUS Corporation, Tokyo, Japan). First, we prepared an observing chamber by punching a 6.0 mm hole in a silicon rubber sheet of 5.0 mm thickness and stuck it on the cover glass. 49 μL of liposomes dissolved in 0.5 M glucose was loaded into the chamber and allowed to settle for at least 15 min to confirm their spherical stability. After the liposomes had settled, 1.0 µL of 50.0 µM peptide dissolved in 0.5 M glucose was gently loaded into the chamber. Thus, the final peptide concentration was 1 μM for each peptide–lipid mixture. To minimize the flow of liposomes, we sunk liposomes near the bottom and observed them in a confined space. The liposomes were observed through a WIG filter (excitation range: 520–550 nm, Hg-lamp). Images were recorded using a DFK33UX252 camera (Argo Corporation, Osaka, Japan). All observations were conducted at RT at least three times. For quantitative analysis, we used Fiji, which is an image-processing package of ImageJ (National Institutes of Health, Bethesda, MD, USA), and Otsu’s threshold clustering algorithm [36].

### 2.3. Image Analysis of Liposomes

In order to evaluate the deformation of liposomes, we conducted a comprehensive analysis comprising three distinct components. The first component, referred to as deformation analysis, involved assessing the frequency with which various peptides were capable of inducing deformation in liposomes. The second component, distortion analysis, focused on quantifying the degree to which each peptide was able to distort liposomes by measuring the average maximum cross-sectional area (Δ*A*_m_) and corresponding time points of five liposomes selected in descending order of aspect ratio (*AR*) after peptide addition. The final component, partial deformation analysis, sought to determine the shape of the local distortion by comparing the partial deformation of individual liposomes before and after peptide addition. To this end, we selected two snapshots within the time-lapse of representative deforming liposomes in each condition: one with the median *AR* before adding peptides to showcase the mean shape of the spherical liposome and another with the Δ*A*_m_ after adding any peptide to demonstrate the distortion.

### 2.4. Measurement of Membrane Capacitance and Subsequent Analysis

Membrane capacitance (*C*_m_) was detected using electrophysiological methodologies. [37] First, we fabricated the body of the detection device to have a 6.0 mm thickness and two separators with 0.2 mm thickness from a polymethyl methacrylate (PMMA) plate using a three-dimensional modeling machine (MM-100, Modia Systems, Saitama, Japan) (Figure S1). Two chambers with a 2.0 mm diameter and a 4.5 mm depth and a groove with a 0.45 mm width between the wells were carved on the device. A hole with a 1.0 mm diameter was carved in each separator. Each chamber in the device had a hole of 0.5 mm diameter in the bottom, and Ag/AgCl electrodes were set into the hole. A polymeric film made of parylene C (polychloro-p-xylylene) with a 5.0 μm thickness was patterned with a single pore of 100 µm diameter using photolithography. The film was sandwiched by two separators and inserted into the groove of the device to separate the two chambers.

Next, 1.5–2.0 µL of 25.4 µM DOPC dissolved in *n*-decane and 5.0 µL of a liquid mixture of 1 µM peptide, 150 mM KCl, and 10 mM MOPS (pH 7) dissolved in MQ were loaded into chambers in order. To contact the aqueous droplets in two chambers, a bilayer was formed at the parylene pore [38,39,40,41,42]. In addition to applying a +5 mV holding voltage, a +5 mV square pulse was given at a pulse frequency of 40 kHz. When the capacitance reached the upper limit of the measurement equipment, we stop the measurement. To assess the effect of peptide binding on *C*_m_, we measured the temporal parameters such as resistance (*R*) and time constant (*τ*) with the capacitance. (See also the result) [43]. All data were recorded with an Axopatch 200B amplifier (Molecular Devices, San Jose, CA, USA) and trials more than n ≥ 3. The recorded data were analyzed with the Clampex 9.0 software (Molecular Devices, San Jose, CA, USA).

## 3. Results

### 3.1. Microscopic Observation of the Deformation and Rupture of Liposomes by Peptides

**Figure 1:**
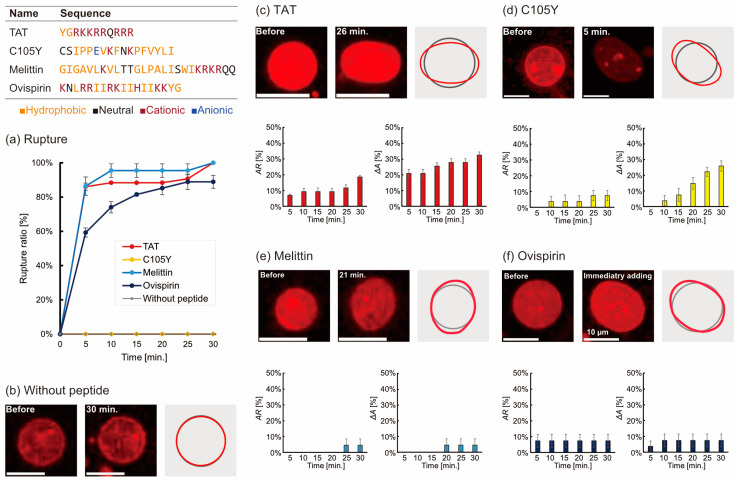
(**a**) The rupture ratio of liposomes during 30 min after adding five peptides. (**b**) Images of liposomes without peptide and (**c**–**f**) with 1 µM peptide addition. The grey line and red line in the right of the images are the tracing outlines of the liposomes before and after adding peptides. The bar graphs show the time dependence of the deforming ratio of *AR* and Δ*A*. The number of trials N ≥ 3 and the number of liposomes n = 10 without peptide and n > 20 with peptide.

Deformation of liposomes was observed through the utilization of fluorescent microscopy for a duration of 30 min following the addition of a peptide at a concentration of 1 µM. To ensure an adequate resolution, only spherical liposomes with a diameter of approximately 6.0 µm were selected; the size distribution is depicted in Figure S2. Upon monitoring the liposomes, it was observed that some liposomes temporarily disappeared due to rupture (as illustrated in Figure S3). When adding TAT, melittin, or ovisprin, more than 80% of liposomes were ruptured within 30 min. In the case of control (without peptide) and C105Y addition, the liposomes were stable, and the rupture was not observed for 30 min (Figure 1a). Liposome deformation was observed in all cases after adding the peptide, and the contour of liposomes fluctuated erratically (distortion), or the size of the liposomes had changed when compared with the control experiment (Figure 1b–f). Liposome deformation was analyzed from two parameters: the temporal change of aspect ratio (*AR*) and the change of cross-sectional area (Δ*A*) as defined below:

(1) $$AR=\frac{L_{l}}{L_{s}}$$

(2) $$\Delta A=\frac{A_{t}}{A_{ave.}}$$

where *L*_s_ and *L*_l_ are the shortest and longest diameter of a given liposome, *A*_t_ is the apparent cross-sectional area at *t* seconds after adding the peptides and *A_ave._* is an average cross-sectional area for a minute before adding peptides, respectively.

Liposomes seem to be slightly deforming without peptides probably due to the convection of the solution. We defined a threshold of large deformation by *AR* and *ΔA* based on the data of the absence of peptides. Deformation of liposomes in this study was defined as over the threshold:

*AR* > 1.1

Δ*A* > 1.1

The *AR* and the Δ*A* provide information on the shape distortion and the size change of liposomes. The order of *AR* at 30 min was as follows (Figure 1c–f):

TAT > C105Y = ovispirin = melittin

The order of Δ*A* was:

TAT > C105Y > ovispirin = melittin

**Figure 2:**
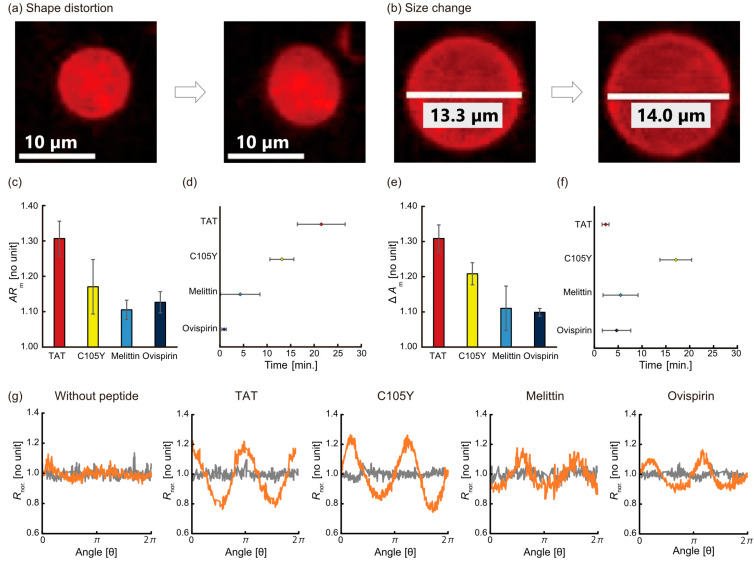
Typical images of the liposome deformation of (**a**) shape distortion and (**b**) size change before and after adding peptides. (**c**) The maximum *AR* (*AR*_m_) in the case of adding each peptide. (**d**) The time of the maximum *AR* (*AR*_m_). (**e**) The maximum Δ*A* (Δ*A*_m_) in the case of adding each peptide. (**f**) The time of maximum Δ*A* (Δ*A*_m_). (**g**) Partial distortion for each condition. The distortions at each angle before (gray) and after adding peptides (orange) were quantified by the deviation of the relative distance from 1.0 indicating the radius of true circle.

We next analyzed these deformation parameters in detail: the distortion estimated by the *AR*, and the size change estimated by the *ΔA* (Figure 2a,b). Regarding the *AR*, the maximum *AR* (*AR*_m_) ranged from 1.1 to 1.4, and TAT showed the highest *AR*_m_ (Figure 2c). The order of *AR*_m_ was:

TAT > C105Y > ovispirin = melittin

The observed *AR*_m_ occurred at different times during the 30 min for each peptide. Melittin and ovispirin reached the *AR*_m_ within 10 min, whereas that of TAT and C105Y were over 10 min (Figure 2d). The time order was similar to the result of *AR* as follows:

TAT > C105Y > melittin > ovispirin

As for the Δ*A*, the maximum Δ*A* (Δ*A*_m_) ranged from 1.1 to 1.4, and TAT also showed the highest Δ*A*_m_ (Figure 2e). The order was similar to the result of *AR* as follows:

TAT > C105Y > melittin = ovispirin

These Δ*A*_m_ were observed before 10 min except for C105Y (Figure 2f). The time order was as follows:

C105Y > melittin ≥ ovispirin > TAT

The quantitative estimation of the distortion was performed by analyzing the angle-dependence radius of liposomes as previously proposed in the literature [3]. The normalized radius (*R_nor._*) was calculated based on an equation:

(3) $$R_{nor.}=\frac{R\left(\theta \right)}{R_{ave.}}$$

where *R_ave._* is the average radius, and *R* (*θ*) is the radius at each angle (*θ*), respectively. The radius of each peptide against the angles is depicted in Figure 2g. The TAT (0.18 ± 0.01) and C105Y (0.15 ± 0.03) system tended to show a higher amplitude of *R_nor_.* than that of the melittin (0.10 ± 0.02) and ovispirin (0.12 ± 0.01) system.

### 3.2. Binding of Peptide on the Lipid Membrane Estimated by the Membrane Capacitance

It has previously been reported that the capacitance of lipid membranes (*C_m_*) increases with the binding of added antimicrobial peptides [44]. In this study, we estimated the binding of peptides TAT, C105Y, melittin, and ovispirin to the lipid membrane by measuring the increase in *C_m_*. These peptides have the ability to form pores or defects during capacitance measurement; thus, we considered the effect of leakage current on *C_m_*. A general capacitance (*C*) is given by the following equation:

(4) C=QV

where *Q* is the charge amount and *V* is the applied voltage.

Leakage current should affect the value of current and resistance if the leaking has occurred. In our experimental system, the temporal current in the circuit (*I*(*t*)) is calculated in the following equation [36]:

(5) $$I_{\left(t\right)}=\frac{V}{R_{m}+R_{s}}\left(1+\frac{R_{m}}{R_{s}}e^{-\frac{t}{\tau }}\right)\;$$

(6) $$\mathrm{where}\;\tau =\frac{R_{m}\;R_{s}\;C_{m}}{R_{m}+R_{s}}$$

In the equations, *R_m_* is membrane resistance, *R_s_* is the series resistance, total resistance except for *R_m_*, *V* is the square pulse voltage, and *τ* is the time constant. *R_s_* and *V* are constant, and *R_m_* and *C_m_* can be affected by the change in current. Equation (6) can be approximated as follow because the *R_m_* >> *R_s_* in our measurements:

(7) $$\tau ≒\frac{R_{m}\cdot R_{s}\cdot C_{m}}{R_{m}}=R_{s}\cdot C_{m}$$

Since *R_s_* is constant, the *C_m_* is proportional to *τ*:

(8) $$\tau \propto C_{m}$$

**Figure 3:**
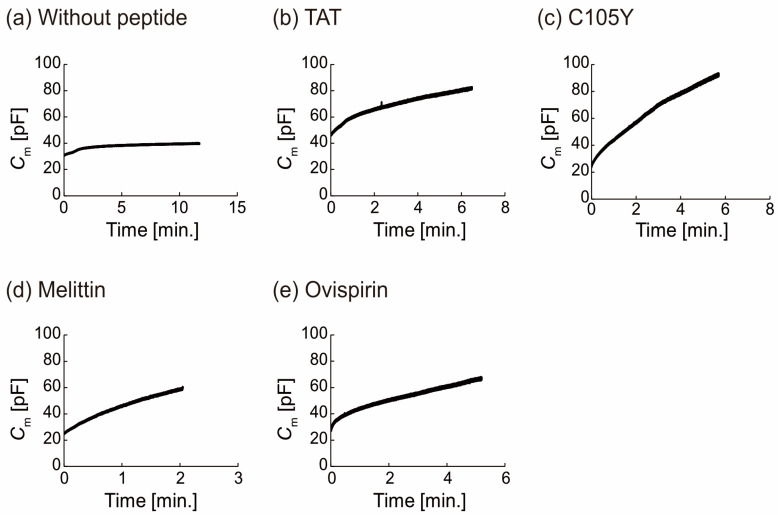
Membrane capacitance measurements for estimating the peptide binding in the lipid membrane. (**a**) without peptide, (**b**–**e**) with peptide.

In order to eliminate the influence of leakage currents on our measurements, we established time zero as the point at which the capacitance (*C_m_*) begins to be proportional to the time constant of current decay (Figure S4). For the purposes of analysis, we restricted our data to that which adhered to Equations (5)–(8), as the purpose was to estimate the change in *C*_m_ resulting from peptide binding, rather than the effect of leakage currents. Our calculations indicate that the impact of leakage currents on the continuous increase in *C_m_* was minimal. In the absence of the peptides, *C_m_* initially increased and then reached a plateau state, as depicted in Figure 3a. This transition from the initial state to the plateau state is indicative of a shift from lipid monolayer to thinner bilayer formation, as observed through the droplet contact method employed in this study [45]. In contrast, in the presence of the peptide, the *C_m_* in the majority of measurements gradually increased from the initial state and did not reach a plateau state, instead reaching the upper limit of the equipment within a matter of minutes (Figure 3b–e). The normalized *C_m_* (*C_nor._*) was calculated in the following equation:

(9) $$C_{nor.}=\frac{C_{max}}{C_{i}}\;$$

where *C_max_* is the maximum *C_m_* and *C_i_* is the initial *C_m_* at the starting time of the measurements (=time 0). The *C_nor._* ranged from 1.3 to 1.5 without peptides and from 1.6 to 4.2 with peptides.

We next estimated that the continuous increase showed the peptide binding. Based on Equation (9), we considered what parameter(s) reflect the increase of the *C_m_*.

The *C_m_* can be defined as:

(10) $$C_{m}=\varepsilon_{0}\varepsilon_{r}\frac{S}{d}$$

where parameters *ε*_0_, *ε_r_*, *S*, and *d* are the permittivity of a vacuum, relative permittivity, surface area, and thickness of the planar bilayer lipid membrane, respectively. The capacitance data allows us to infer that peptides are binding to the membrane, as the parameters can vary in response to peptide binding. The variations in the effective lipid bilayer area (*S*) and the thickness of the membrane are expected to be minimal in comparison to the changes in *ε_r_* as the *ε_r_* value of peptides (around 54) [46] is significantly greater than that of DOPC molecules (around 2 to 3) (Figure S5) [47]. Therefore, we consider that the increase in *C_m_* is due to peptides binding to the lipid membrane and an increase in peptide binding resulting in the *ε_r_* increase. In addition, the *ε_r_* change of the four peptides was similar (around 40 pF, n ≥ 3, the control was ca. 10 pF, n = 3), indicating a comparable amount of bindings to the lipid membrane.

## 4. Discussion

The ability of four peptides, TAT (CPP), C105Y (CPP), melittin (AMP), and ovispirin (AMP), to induce liposome deformation was investigated utilizing fluorescence microscopy and membrane capacitance measurements. The findings of this study revealed that C105Y did not exhibit the ability to rupture liposomes, unlike the other three peptides, which caused the disappearance of over 70% of liposomes within 10 min. The amphipathicity of peptides was found to be a significant factor in the ability to rupture liposomes. [48] C105Y (17 aa.) has a Glu and two Lys at the 6th, 8th, and 11th positions, and its charge distribution sequence imparts a relatively low degree of amphipathicity [24,26]. In contrast, the other three peptides possess relatively stronger amphipathicity due to their amphipathic α-helical structure (melittin and ovispirin) and charge polarization structure (TAT).

We next found that the capability of liposome deformation used in TAT and C105Y was greater than that in melittin and ovispirin. Initially, it was hypothesized that this difference was due to variations in the amount of peptide binding to the lipid membrane. However, subsequent membrane capacitance measurements revealed that the binding of peptides was almost equivalent among the peptides. Therefore, the observed difference in deformation may be attributed to variations in the secondary structure of the peptides, which leads to distinct interactions and localization within the lipid membrane. Peptides with random coil structures display random-like behavior within the lipid membrane, [24,25], and it has been proposed that conventional CPPs bind to lipid membranes and exhibit behavior akin to that of detergents or form inverted micelle structures. [24,25]. In contrast, peptides with an α-helical structure have amphiphilic properties upon binding to the surface of the lipid membrane and tend to form a transmembrane structure or cover the surface in a carpet-like manner [19,28,29,30,31,32,33,34]. This hypothesis is supported by molecular dynamics simulation (see also supporting description and Figure S6) [49,50,51,52,53,54]. The secondary structure and the membrane-binding mode of these peptides have a significant impact on macroscopic membrane deformation in liposomes.

## 5. Conclusions

In conclusion, we investigated the ability of four distinct peptides, CPPs and AMPs, to induce liposome deformation. The deformation and rupture of liposomes were monitored using fluorescent microscopy. It was observed that C105Y did not induce liposome rupture and that the degree of deformation and shape distortion varied between TAT and C105Y (CPPs) and melittin and ovispirin (AMPs). The binding of peptides to the lipid membrane was estimated using membrane capacitance (*C_m_*) measurements, which indicated that there was not a significant difference in the amount of peptide binding among the peptides. These variations in the effects on liposome deformation may be attributed to variations in the secondary structures of the peptides when bound to a DOPC membrane. Our studies provide insight into the utilization of peptides to control liposome shape and offer a promising approach for regulating the bodies of liposomal molecular robots. In future research, we will design peptides capable of controlling liposome deformation, incorporate these peptides into liposomes, and modulate liposome deformation depending on the context.

## Data Availability

Not applicable.

## Supplementary Materials

The following supporting information can be downloaded at: https://www.mdpi.com/article/10.3390/mi14020373/s1, Figure S1: the device of capacitance measurement; Figure S2: size distribution of analyzed liposomes; Figure S3: rupture of liposomes; Figure S4: the principle of capacitance detection; Figure S5: correlation between physical parameters and physical parameters at the membrane; Figure S6: all-atom MD simulation for the migration of 400 ns
