# Supplementary material for: Liposome Deformation Induced by Membrane-Binding Peptides
Source: Micromachines (Basel). 2023 Feb 2;14(2):373. doi: 10.3390/mi14020373 (PMC9967443; doi:10.3390/mi14020373)
Supplement: Supplementary file 1 [file micromachines-14-00373-s001.zip › micromachines-2153257-SI.pdf]

Liposome Deformation Induced by Membrane-Binding Peptides

Kayano Izumi, Chihiro Saito and Ryuji Kawano

Department of Biotechnology and Life Science, Tokyo University of Agriculture and Technology, Tokyo 184-8588, Japan

KEYWORDS Liposome deformation, Transmembrane peptides, Membrane capacitance, Molecular dynamic simulation

Molecular dynamics simulation of membrane binding peptides in lipid membrane

We used molecular dynamics (MD) simulations to investigate the effect of the peptide localization on liposome deformation because the detail-localization dynamics in the membrane are difficult to be evaluated by direct observation like the localization of fluorescently labeled peptides. All-atom MD simulations were performed using GROMACS 2021.22 [1] and the CHARMM36mforce field [2]. The initial coordinates of TAT and melittin were taken from structures obtained by x-ray diffraction or nuclear magnetic resonance (PDB codes 1TBC and 2MLT). Coordinate models in which a peptide was placed in a bilayer of 200 DOPC molecules were prepared by the CHARMM-GUI server [3] . The prepared simulation boxes were filled with TIP3 water. For neutralization, we added OH^- as counterions of peptides. All simulations were first run using the steepest descent algorithm to minimize energy and then equilibrate temperature (298.15 K) and pressure (1 bar). Overall temperature and pressure control were carried out with the Nose-Hoover thermostat and Parrinello-Rahman barostat [4], respectively. All systems were performed under periodic boundary conditions in all directions. For short-range van der Waals and electrostatic cutoffs, the default optimized distance of 1.2 nm was used and the force switches potential modifier was applied from 1.0 to 1.2 nm. Long-range electrostatic interactions were calculated using the particle mesh Ewald (PME) algorithm [5]. The hydrogen-related bonds were constrained by the linear constraint solver (LINCS) algorithm [6] . Finally, 400 ns of simulations were performed using the leapfrog algorithm. Analyses were performed using the GROMACS package. Visualizations were performed using PyMoL.

We attempt to discuss the difference by focusing on the difference in secondary structures. The secondary structure of the peptides in lipid membranes can be categorized as either random coil (CPPs: TAT and C105Y) or α-helix (AMPs: melittin, and ovispirin). The random coiled peptides (TAT) did not have a fixed configuration in the lipid membrane and randomly moved to the one leaflet of the membrane (**Figure S6a, c**) while the α-helical peptide (melittin) tended to show a perpendicular structure and kept the helicity (**Figure S6b, d**).

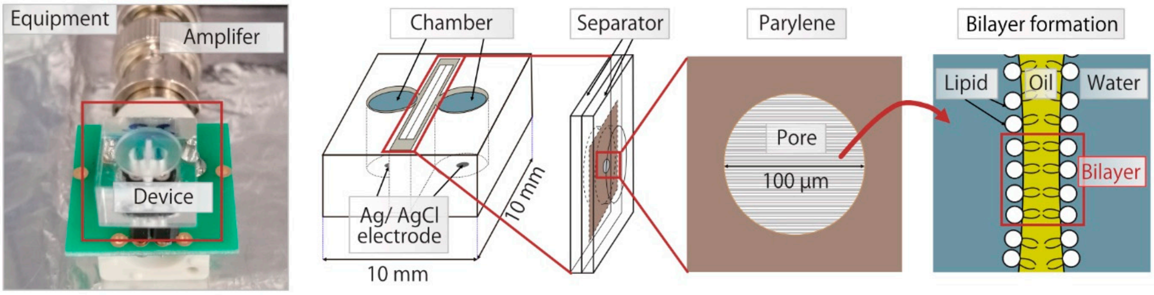

Figure S1 The device of capacitance measurement We made the bilayer lipid membrane at a 100 μm parylene pore.

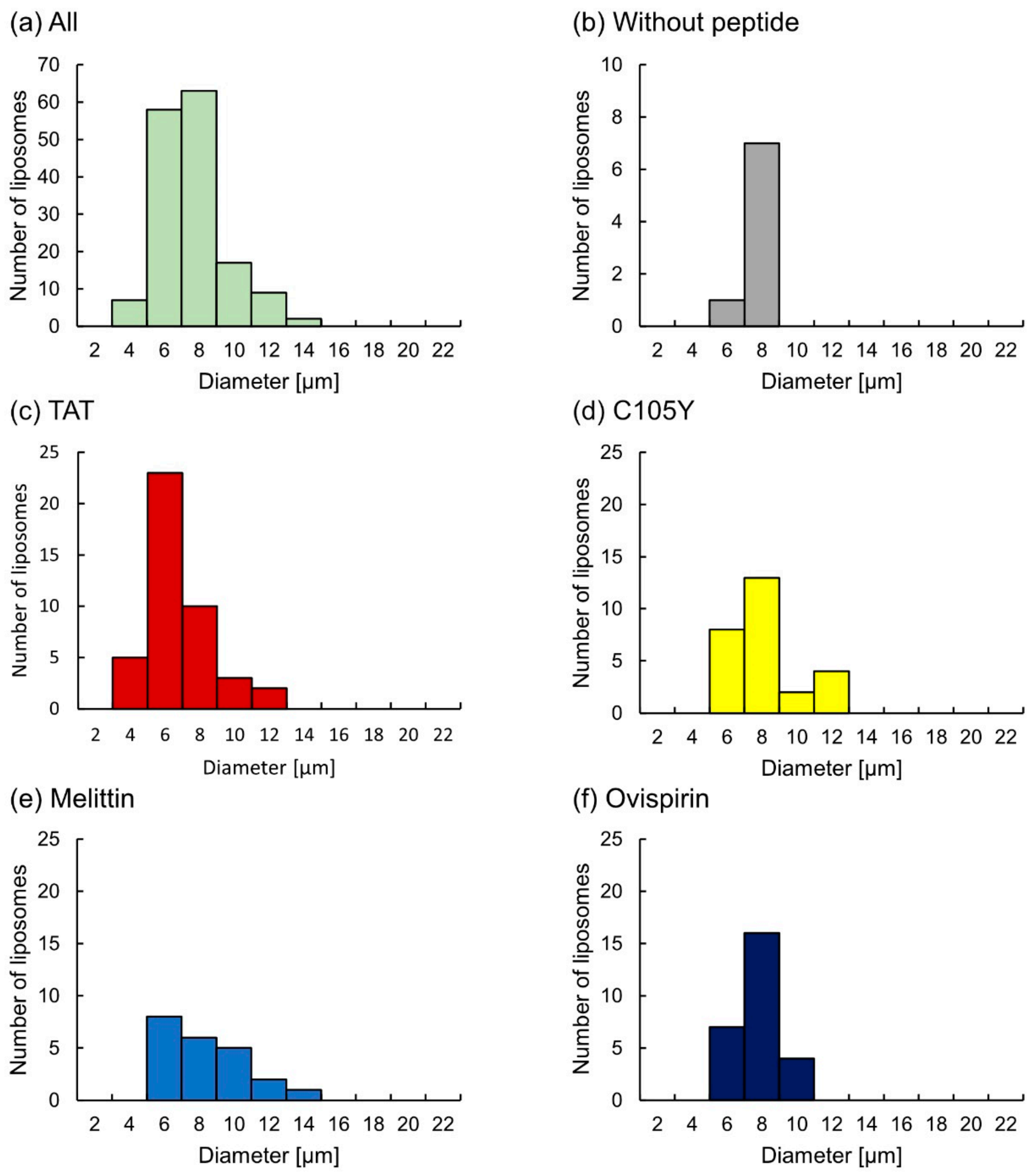

Figure S2 Size distribution of analyzed liposomes (a) the liposome distribution in all experimental conditions, and (b-f) the liposome distribution at each conditions. To ensure the analytical resolution, we selected liposomes whose diameters are more than 6 μm in chamber.

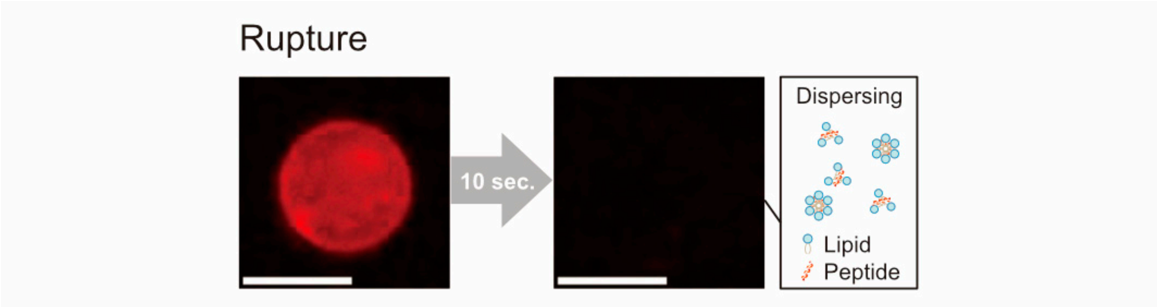

Figure S3 Rupture of liposomes Some liposomes adding peptides ruptured during the observation.

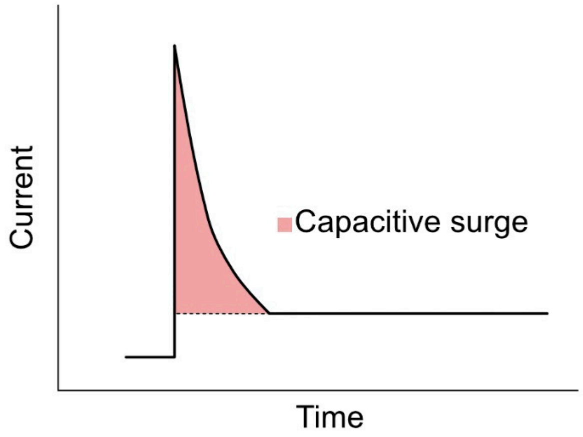

Figure S4 The principle of capacitance detection We took part in consideration the current region in red.

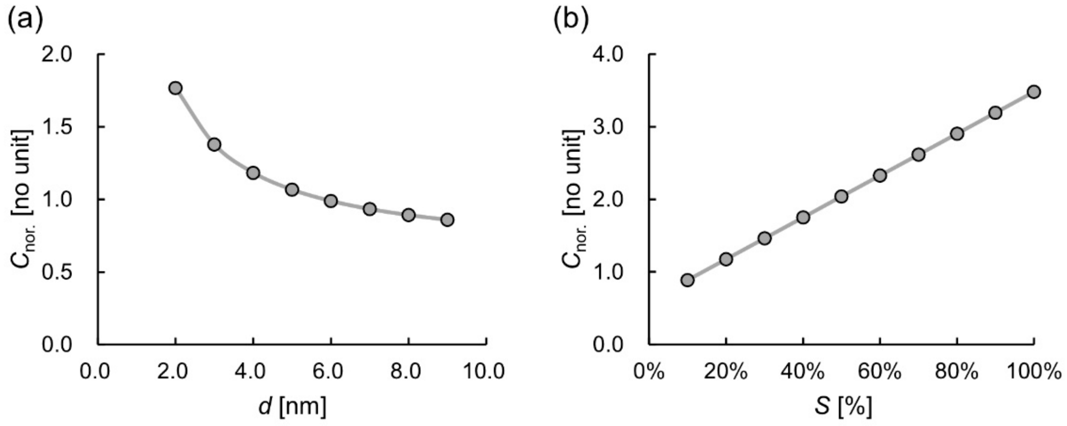

Figure S5 Correlation between physical parameters and physical parameters at the membrane (a) The change of the effective membrane thickness (*d*). The relative permittivity (ε_r) and the effective lipid bilayer area (*S*) were fixed 2.8 and 30% against the area of parylene pore (diameter: 100 μm) respectively. (b) The change of the *S*. The ε_r *d* were fixed 2.8 and 2.7 nm respectively.

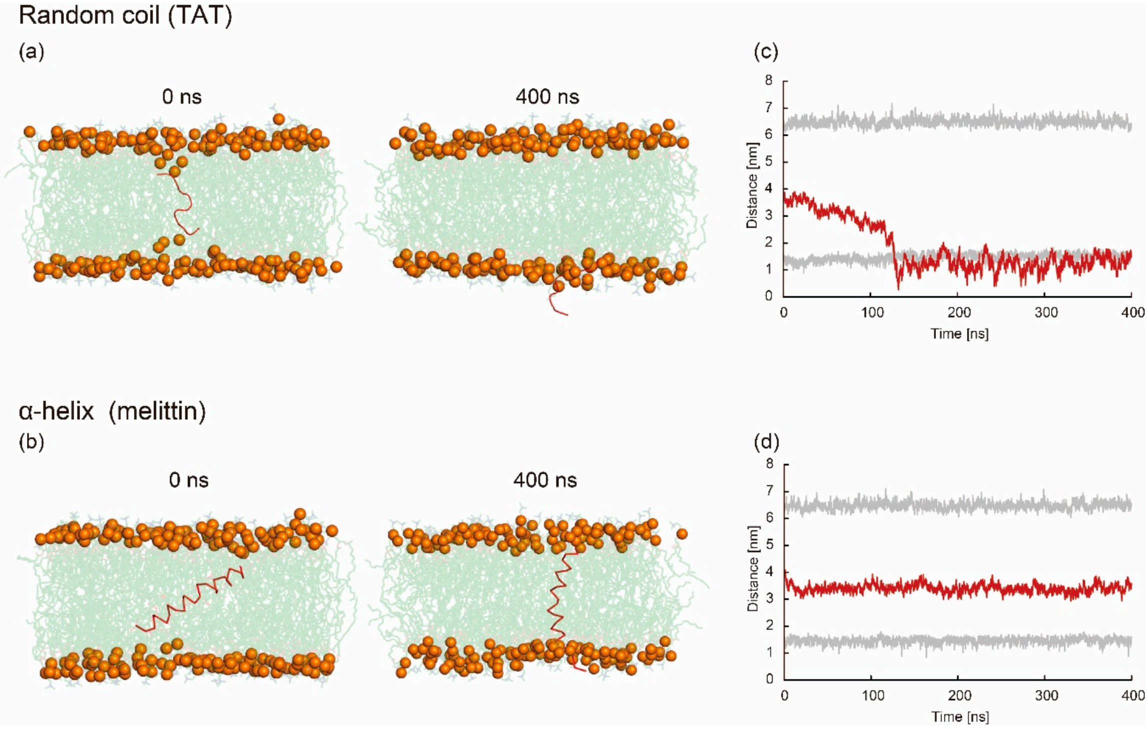

Figure S6 All-atom MD simulation for the migration of 400 ns (a) Random coil and (b) α-helix peptides are in a lipid membrane. For understanding the migration, we drawn the peptides as alpha-carbon chain. (c) (d) Relative locational relationship between each peptide and lipid membrane. The graph plots show temporal location average location of lipid head group for each layer (grey) and each peptide (red).

1. Van Der Spoel, D.; Lindahl, E.; Hess, B.; Groenhof, G.; Mark, A.E.; Berendsen, H.J. GROMACS: fast, flexible, and free. *Journal of Computational Chemistry* **2005**, *26*, 1701-1718, doi:10.1002/jcc.20291.
2. Huang, J.; Rauscher, S.; Nawrocki, G.; Ran, T.; Feig, M.; de Groot, B.L.; Grubmüller, H.; MacKerell, A.D., Jr. CHARMM36m: an improved force field for folded and intrinsically disordered proteins. *Nat Methods* **2017**, *14*, 71-73, doi:10.1038/nmeth.4067.
3. Jo, S.; Kim, T.; Iyer, V.G.; Im, W. CHARMM-GUI: a web-based graphical user interface for CHARMM. *Journal of Computational Chemistry* **2008**, *29*, 1859-1865, doi:10.1002/jcc.20945.
4. Parrinello, M.; Rahman, A. Polymorphic transitions in single crystals: A new molecular dynamics method. *Journal of Applied Physics* **1981**, *52*, 7182-7190, doi:10.1063/1.328693.
5. Essmann, U.; Perera, L.; Berkowitz, M.L.; Darden, T.; Lee, H.; Pedersen, L.G. A smooth particle mesh Ewald method. *The Journal of Chemical Physics* **1995**, *103*, 8577-8593, doi:10.1063/1.470117.
6. Hess, B.; Bekker, H.; Berendsen, H.J.C.; Fraaije, J.G.E.M. LINCS: A linear constraint solver for molecular simulations. *Journal of Computational Chemistry* **1998**, *18*, 1463-1472, doi:10.1002/(SICI)1096-987X(199709)18:12<1463::AID-JCC4>3.0.CO;2-H.
